# E3 Ubiquitin Ligase UBR5 Promotes the Metastasis of Pancreatic Cancer *via* Destabilizing F-Actin Capping Protein CAPZA1

**DOI:** 10.3389/fonc.2021.634167

**Published:** 2021-03-12

**Authors:** Jin Li, Wei Zhang, Jian Gao, Min Du, Huimin Li, Mengge Li, Hui Cong, Yuan Fang, Yiyi Liang, Dan Zhao, Gang Xiang, Xiaojing Ma, Ming Yao, Hong Tu, Yu Gan

**Affiliations:** ^1^State Key Laboratory of Oncogenes and Related Genes, Shanghai Cancer Institute, Renji Hospital, Shanghai Jiao Tong University School of Medicine, Shanghai, China; ^2^Department of Medical Oncology, The First Affiliated Hospital of USTC, Division of Life Sciences and Medicine, University of Science and Technology of China, Hefei, China; ^3^Organ Transplantation Center, The First Affiliated Hospital of Kunming Medical University, Kunming Medical University, Kunming, China; ^4^State Key Laboratory of Microbial Metabolism, Sheng Yushou Center of Cell Biology and Immunology, School of Life Science and Biotechnology, Shanghai Jiao Tong University, Shanghai, China

**Keywords:** UBR5, metastasis, CAPZA1, ubiquitination, pancreatic cancer

## Abstract

The ubiquitin-proteasome system (UPS) is a regulated mechanism of intracellular protein degradation and turnover, and its dysfunction is associated with various diseases including cancer. UBR5, an E3 ubiquitin ligase, is emerging as an important regulator of the UPS in cancers, but its role in pancreatic cancer is poorly understood. Here, we show that UBR5 is significantly upregulated in pancreatic cancer tissues. High UBR5 expression is correlated with increased lymph node metastasis and poor survival of patients. The loss-of-function and gain-of-function studies demonstrated that UBR5 substantially enhanced the *in vitro* migratory and invasive ability of pancreatic cancer cells. UBR5 knockdown also markedly inhibited *in vivo* cancer metastasis in the liver metastatic model of pancreatic cancer in nude mice, suggesting UBR5 as a potent metastatic promoter in pancreatic cancer. Furthermore, using co-immunoprecipitation combined with mass spectrometry analyses, CAPZA1, a member of F-actin capping protein α subunit family, was identified as a novel substrate of UBR5. UBR5 overexpression could promote the degradation of CAPZA1 via the UPS and induce the accumulation of F-actin, which has been described as an essential molecular event during the process of CAPZA1 deficiency-induced cancer cells migration and invasion. UBR5 knockdown significantly increased the intracellular level of CAPZA1 and CAPZA1 downregulation largely reversed the UBR5 knockdown-induced suppression of cell migration and invasion in pancreatic cancer cells. Collectively, our findings unveil UBR5 as a novel and critical regulator of pancreatic cancer metastasis and highlight the potential for UBR5-CAPZA1 axis as a therapeutic target for preventing metastasis in pancreatic cancer patients, especially in those with increased UBR5 expression.

## Introduction

Pancreatic cancer is the fourth leading cause of cancer-related death (1) and the average survival for pancreatic cancer patients is only 6–9 months after diagnosis (2). More importantly, pancreatic cancer patients are often diagnosed in advanced stages when the cancer has spread to distant sites. It's estimated that approximately 80–85% of pancreatic cancer patients have died due to metastasis (3, 4). Therefore, it is urgent to clarify the mechanisms involved in pancreatic cancer metastasis.

The ubiquitin-proteasome system (UPS) is a major posttranslational mechanism of intracellular protein degradation and turnover in eukaryotes (5, 6). Abnormal UPS function has been observed in various pathological states (7). In recent years, the role of UPS in cancer metastasis is gaining more and more interest (8). Protein ubiquitination is multistep process that is mediated by ubiquitin activating enzymes (E1s), ubiquitin-conjugating enzymes (E2s), and ubiquitin ligases (E3s), respectively (9). It was found that the E2 ubiquitin-conjugating enzyme UbcH10 or UBE2Q1 functioned as an oncogene to facilitate metastasis in colorectal cancer (10) or hepatocellular carcinoma (11). These findings indicate the importance of UPS in cancer metastasis and studies may provide insight into the mechanism underlying metastatic progression.

UBR5 is one of the E3 ligases, which occupy a key position in the UPS enzymatic cascade because they largely determine the specificity of the protein substrate to be ubiquitinated (12). It was indicated that UBR5 had a wide range of interacting protein partners, which are known to be implicated in multiple cellular processes such as DNA damage and cell cycle regulation (13, 14). For instance, UBR5 was shown to regulate transcription via promoting the ubiquitination-mediated degradation of E3 ligase RNF168 at damaged chromatin (13). UBR5-mediated ubiquitination and degradation of its interacting proteins was subsequently demonstrated to play an important role in these critical cellular processes.

Emerging studies have linked UBR5 to cancers recently. UBR5 was originally identified as a cancer-related gene in a screening for progestin-regulated genes in breast cancer cells (5). Provisional data from The Cancer Genome Atlas (TCGA) reveal UBR5 gene amplification as a common genetic alteration in various cancer types (15). Consistently, UBR5 was shown to be significantly upregulated in various cancer samples such as gastric cancer and breast cancer (16–20). High UBR5 expression was associated with adverse prognosis in breast cancer and gallbladder cancer (19, 21). The tumor-promoting effect of UBR5 has been largely attributed to its ubiquitin ligase activity. In colorectal and gastric cancers, UBR5 was found to accelerate tumor growth by destabilizing the tumor suppressors ECRG4 and GKN1 following their ubiquitination (16, 22). These findings suggested UBR5 as a vital regulator of UPS in cancers. However, its biological functions, as well as the underlying mechanisms, in pancreatic cancer still are poorly understood.

In this study, UBR5 was found to be upregulated in pancreatic cancer tissues and the increased UBR5 expression level was significantly associated with both lymph node metastasis and the poor survival of patients. UBR5 was then demonstrated to promote cancer metastasis in both *in vitro* and *in vivo* models of human pancreatic cancer. We further identified F-actin-capping protein subunit alpha-1 (CAPZA1) as a novel substrate of UBR5 and described a CAPZA1–related mechanism underlying the metastasis-promoting effect of UBR5 in pancreatic cancer.

## Materials and Methods

### Public Database

The mRNA expression patterns of UBR5 in multiple cancers and normal tissues were obtained from the web-based Oncomine (https://www.oncomine.org/resource/login.html) database. Comparison of UBR5 mRNA expression in pancreatic cancer samples and the normal pancreatic samples from The Cancer Genome Atlas (TCGA) and the Genotype-Tissue Expression (GTEx) databases was performed using the web-accessible expression analysis tool from Gene Expression Profiling Interactive Analysis (http://gepia.cancer-pku.cn/) (23). The mRNA expression of UBR5 in pancreatic cancer tissues compared with noncancerous pancreatic tissues was analyzed using data from the Gene Expression Omnibus (GEO) (https://www.ncbi.nlm.nih.gov/geo/). The evaluation of UBR5 protein expression in solid cancers from the Clinical Proteomic Tumor Analysis Consortium (CPTAC) dataset was performed using UALCAN analysis tool (http://ualcan.path.uab.edu/analysis.html). The overall and relapse-free survival curves of pancreatic cancer patients from the TCGA dataset were generated by the online survival analysis tool Kaplan–Meier plotter (http://kmplot.com/analysis/) (24). The UBR5 high expression and low expression groups were classified using the “Auto select best cutoff” function. The survival curves were calculated using the Kaplan–Meier method and analyzed by the log-rank Mantel-Cox test.

### Cell Culture

The human pancreatic cancer cell lines (AsPC-1, BxPC-3, PANC-1, MIA PaCa-2, and CFPAC-1), embryonic kidney cells (HEK-293T) and the human normal pancreatic epithelial cell line (hTERT-HPNE) were purchased from the American Type Culture Collection (ATCC, USA). Cells were cultured in DMEM (MIA PaCa-2, PANC-1, hTERT-HPNE), RPMI-1640 (BxPC-3, AsPC-1) or IMDM (CFPAC-1) medium supplemented with 10% FBS in a humidified atmosphere of 5% CO_2_ at 37°C. All cell lines were authenticated by STR profiling by Biowing Applied Biotechnology Ltd (Shanghai, China).

### Tissue Microarray and Immunohistochemistry

To analyze the UBR5 protein expression in pancreatic cancer tissues, a commercially available tissue microarray containing 29 pairs of pancreatic cancer tissues and the adjacent non-tumorous specimens, which were collected by National Engineering Center For Biochip at Shanghai from Taizhou Hospital from 2017 to 2018 (Approved by Research Ethics Committee of Taizhou Hospital), was used for the immunohistochemical staining of UBR5. All patients had not received adjuvant radiotherapy and chemotherapy before surgery. The clinical staging for pancreatic cancer ranges from stage I through IV. The liver tissues from nude mice with metastatic tumors were resected, fixed in 4% phosphate-paraformaldehyde (for at least 72 hours), embedded in paraffin and sectioned at 5 μm. The sections were performed using IHC as well as hematoxylin-eosin (H&E) staining. Briefly, the procedures for deparaffinization, rehydration, antigen retrieval and blocking were carried out as described previously (25). The sections were incubated with the primary antibodies at 4°C overnight and treated with the secondary antibodies for 30 min at room temperature. The information about antibodies is shown in Supplementary Table 3. The primary antibodies were detected using the VECTOR NovaRED (Vector Laboratories, SK-4800). The intensity of the immunostaining was evaluated as follows: negative, 0 point; weak, 1 point; moderate, 2 points; strong positive, 3 points. The percentage of positive tumor cells was scored as 0 (< 5%), 1 (< 25%), 2 (25–50%), 3 (51–75%), and 4 (> 75%). The final scores (1 to 12) were based on the percent of positive cells and staining intensity.

### Lentiviral Infection

To knock down UBR5, CFPAC-1 and PANC-1 cells were infected with the lentivirus (MOI:10) expressing shRNAs, which was constructed and packaged by the Shanghai Genechem Ltd (Shanghai, China), and then were selected with 1μg/ml puromycin (Gibco, A11138-03) for 7–10 days (26). A scrambled shRNA was used as the negative control (shNC). The sequences targeting UBR5 are listed in Supplementary Table 1.

### Transient Transfection

The pCDH-GFP (vector control) plasmid was constructed by inserting GFP into pCDH-CMV-MCS-EF1-puro between Xbal and BamHI sites and pCDH-GFP-UBR5 (GFP-tagged UBR5) plasmid was constructed by cloning the human UBR5 gene into pCDH-GFP between BstBI and NotI sites. The CAPZA1 siRNAs were supplied by Guangzhou RiboBio Ltd (Guangzhou, China). The target sequences of CAPZA1 siRNA are listed in Supplementary Table 1. To construct the UBR5 overexpression cells, 18 μg vector control or GFP-tagged UBR5 plasmid was transfected into 5 × 10^6^ MIA PaCa-2, BxPC-3 or HEK-293T cells via electroporation using NucleofectorTM 2b Device (Lonza, #AAB-1001). To silence CAPZA1, the siRNAs were transfected into CFPAC-1 and PANC-1 cells infected with shUBR5 using Lipofectamine 3000 reagent (Invitrogen, L3000015) according to the manufacturer's instruction. 72 h after transfection, the cells were harvested and subjected to subsequent experiments.

### Quantitative Real-Time PCR

To detect gene expressions at the mRNA level, total RNA of cells was isolated using TRIzol reagent (Life technologies, 15596018) and cDNA was synthesized using 2μg of total RNA with PrimeScriptTM RT Master Mix (Takara, #RR036ART). The quantitative real-time PCR was performed using the FastStart Universal SYBR Green kit (Roche, 049139) on StepOne Plus Real-Time PCR System (Applied Biosystems). The housekeeping gene, *GAPDH*, was used as an internal control. Relative mRNA expression levels of genes were analyzed by the 2^(−ΔΔCt)^ method. The quantitative real-time PCR primers used in this study are listed in Supplementary Table 2.

### Western Blot Analysis

To analyze gene expressions at the protein level, cells were lysed in cold RIPA buffer freshly supplemented with 1 mM phenylmethylsulfonyl fluoride (PMSF). The BCA Protein Assay Kit (Thermo Fisher Scientific, 23227) was used to measure the protein concentration. Protein extracts were separated by sodium dodecyl sulfate-polyacrylamide gel (SDS-PAGE) and transferred onto polyvinylidene fluoride membrane (Millipore, IPVH00010). After blocked with 5% non-fat milk, the membrane was stained with the corresponding primary antibodies at 4°C overnight and subsequently incubated with HRP-conjugated secondary antibodies for 1 hour. *GAPDH* as an internal control was utilized. The information about antibodies is shown in Supplementary Table 3. All western blot bands were quantified using Image J software and the quantitative results were presented as the relative expression levels of target proteins normalized to the corresponding internal controls.

### Wound Healing and Invasion Assays

The wound healing assays were carried out to examine cell migration ability. The confluent cell monolayers were scratched using 20 μl pipette tips and then cultured for 12–36 h depending on cell types. Cell invasion assays were performed with 8.0 μm filter chambers (Corning, 353097). The upper chamber was coated with the serum-free culture medium-diluted Matrigel (Corning, 356234) and then 1 × 10^5^ CFPAC-1, PANC-1, MIA PaCa-2 and BxPC-3 cells in 200 μl serum-free culture media were placed into the upper chambers, respectively. At the same time, 700 μl of complete medium was added in the lower chamber. The cells in the bottom of the chamber were fixed with 100% methanol for 10 min, and subsequently stained with 0.2% crystal violet for 30 min, after incubation in a humidified incubator under 5% CO_2_ for 12–36 h. The positively stained cells were captured and counted.

### Animal Experiments

To evaluate the metastasis potency of UBR5 *in vivo*, we established the intrasplenic injection model. CFPAC-1 cells infected with shNC and shUBR5 (1 × 10^6^ cells in 50 μl PBS) were injected into the spleen of male nude mice (6-week-old) as described previously (27). After 6 weeks, the mice were sacrificed under deep anesthesia. The number of metastatic tumors was counted and the size of the largest tumor per liver was measured. All modeling procedures and experimental operations were approved the Medical Experimental Animal Care Commission at the Shanghai Cancer Institute.

### Co-immunoprecipitation

HEK-293T were transfected with the vector control or the GFP-tagged UBR5 plasmid. Cells were treated with proteasome inhibitor MG132 (Selleck, 2619) for 6 hours before harvesting. To reduce the non-specific binding, total lysate (up to 1.5 mg per sample) was incubated with 30 μl protein A/G agarose beads (SANTA CRUZ, #C0620) for 4 h. The agarose beads were removed and then 4 μg primary antibody (anti-GFP or anti-CAPZA1 antibody) was added at 4°C overnight. Next, 30 μl protein A/G agarose beads were added to each immunoprecipitation mixture for 4 h. Subsequently, the precipitates were washed for 4 times and boiled in 2 × loading buffer for 5 min. The immune precipitates were separated by SDS-PAGE and then stained with the Silver Staining Kit (Beyotime, P0017S). The entire two lanes were excised and subjected to mass spectrometry analysis.

### Mass Spectrometry

The mass spectrometry analysis was performed by the Luming Biotechnology Ltd (Shanghai, China) with following methods: Briefly, the entire gel was eluted by dehydration buffers, digested with digestion buffer and desalted. Desalted peptides were analyzed by the Q-Exactive HF mass spectrometer (Thermo Scientific, USA) attached to the Easy-nLC 1200 system (Thermo Scientific, USA) running at 300 nl/ min for 60 min using a three-step acetonitrile gradient: 0–30% over the first 55 min and 30–50% for 80 min and 100% for 80–90 min. Data dependent acquisition mode with a 10 MS2 scan was enabled. The MS1 survey scan (300–1,600m/z) was at a resolution of 35,000 at 200 m/z with automatic gain control (AGC) of 1e6 and a maximum injection time of 50 ms. Dynamic exclusion time was 60.0 s. Each full scan takes 20 MS2 scans. MS2 scan was at a resolution of 17,500 at 200 m/z with automatic gain control (AGC) of 2e5. The RAW files generated by spectrometer was uploaded to ProtemeDiscover 2.4 software for protein identification. The parameters for identification were set as follows: MS1 tolerance, 10 ppm; MS2 tolerance, 0002Da; Missed cleavage, 2; Static modification, carbamidomethyl (C); Dynamic modification, Acetyl (Protein N-term), Deamidated (NQ), Oxidation (M).

### Immunofluorescent Confocal Imaging

Cells cultured on the coverslips were fixed with 4% paraformaldehyde for 20 min and permeabilized with 0.2% Triton X-100 for 10 min. After washed by PBS for 3 times, cells were blocked with 5% BSA for 30 min and incubated with the primary antibodies at 4°C for 12–16 h. Subsequently, the glass slides were incubated with secondary antibodies for 30 min at 37°C in the dark room. The nuclear was stained with DAPI in PBS for 15 min. The information about antibodies is shown in Supplementary Table 3. Cells Images were captured using a Leica TCS SP8 confocal system.

### Statistical Analysis

The statistical analyses were performed with GraphPad Prism 5.0 (GraphPad Software Ltd, San Diego, CA) and presented as the mean ± SEM. The Shapiro-Wilk test was used to test the normality of data distribution. The significance of the differences between groups was assessed using the Student's *t* test (for the normally distributed variables) or the Wilcoxon rank sum test (for the non-normally distributed variables). Correlations between UBR5 expression levels and clinicopathological features in pancreatic cancer cases from TMA and TCGA cohorts were analyzed using the Chi-square test. The level of significance was set at 0.05 for all of the analyses.

## Results

### UBR5 Is Upregulated in Pancreatic Cancer and Associated With Poor Prognosis

**Figure 1:**
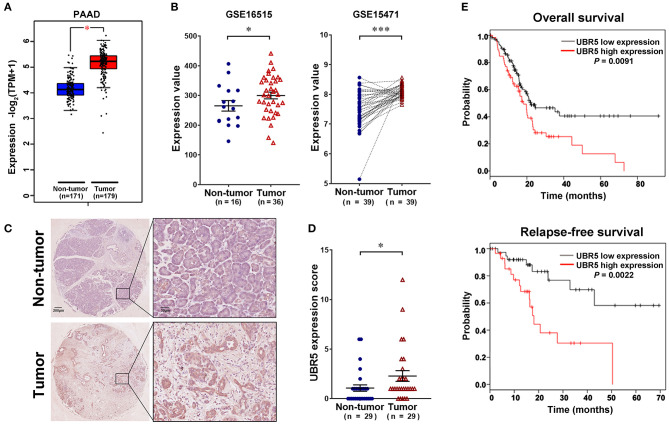
UBR5 is upregulated in pancreatic cancer and associated with poor prognosis. **(A)** The analysis of UBR5 mRNA expression in 171 non-tumor pancreatic tissues and 179 pancreatic cancer tissues. **(B)** The mRNA expression of UBR5 from the GEO database. Representative images **(C)** and quantification of UBR5 staining score **(D)** in 29 paired pancreatic cancer tissues. A paired Student's *t* test was used to compare the expression of UBR5 between pancreatic cancer tissues and normal tissues. **(E)** The overall survival and relapse-free survival analysis of pancreatic cancer patients based on the data from TCGA database using the web-accessible Kaplan–Meier plotter. Survival curves were calculated using the Kaplan–Meier method and analyzed by the log-rank test. *, *P* < 0.05; ***, *P* < 0.001.

To gain initial insight of UBR5 expression patterns, we firstly analyzed data retrieved from the web-based Oncomine database. It was shown that UBR5 mRNA overexpression was present in most human cancers compared with normal tissues, including pancreatic cancer (Supplementary Figure 1). CPTAC data also indicates that the protein expression level of UBR5 was upregulated in various solid cancers (Supplementary Figure 1). Then we focused on pancreatic cancer and found that the UBR5 mRNA expression was remarkably increased in pancreatic cancer tissues compared with noncancerous pancreatic tissues based on the RNA-seq data from The Cancer Genome Atlas (TCGA) and the Genotype-Tissue Expression (GTEx) databases, as well as the microarray-based gene expression profiles (GSE16515 and GSE15471) from GEO database (Figures 1A,B). The protein expression of UBR5 was examined parallelly using immunohistochemical staining on tissue microarray (TMA). Consistently, the protein level of UBR5 was remarkably increased in pancreatic cancer tissues compared with adjacent noncancerous tissues (Figures 1C,D). We then classified pancreatic cancer patients from TMA cohort and TCGA into the low and high expression groups according to the expression of UBR5 and analyzed the correlation between clinicopathological features and UBR5 expression level. The results suggested that UBR5 was remarkably associated with lymph node metastasis and tended to be associated with pathological tumor stage (Table 1). In addition, online survival analysis exhibits patients with high UBR5 expression have significantly shorter overall survival (HR = 1.71 (1.14–2.58), *P* = 0.0091) and relapse-free survival (HR = 3.6 (1.5–8.63), *P* = 0.0022), compared with those carrying low UBR5 expression (Figure 1E). Collectively, these data suggest a vital role of UBR5 in pancreatic cancer.

**Table 1:** Pooled analysis of the correlation between UBR5 expression and clinicopathologic features in pancreatic cancer cases from TMA and TCGA cohorts.

**Clinicopathologic**	**Low**	**High**	**OR (95%) CI**	***P*-value**
**features**	**(*n* = 76)**	**(*n* = 59)**		
**Gender**				
Male Female	45 31	34 25	0.997(0.477–2.084)	0.993
**Median age**				
<60 ≥60	26 50	18 41	1.351(0.626–2.914)	0.443
**pT status**				
T_1_/T_2_ T_3_/T_4_	18 58	5 54	2.590(0.864–7.763)	0.089
**pN status**				
Absence (N_0_) Presence (N_1−3_)	30 46	9 50	3.761(1.535–9.219)	0.004
**Location**				
Head Body/tail	42 34	55 4	0.408(0.169–0.988)	0.047

*Pt, tumor pathological stage; pN, pathological nodes; TMA, tissue microarray*.

### UBR5 Promotes Pancreatic Cancer Migration and Invasion *in vitro*

**Figure 2:**
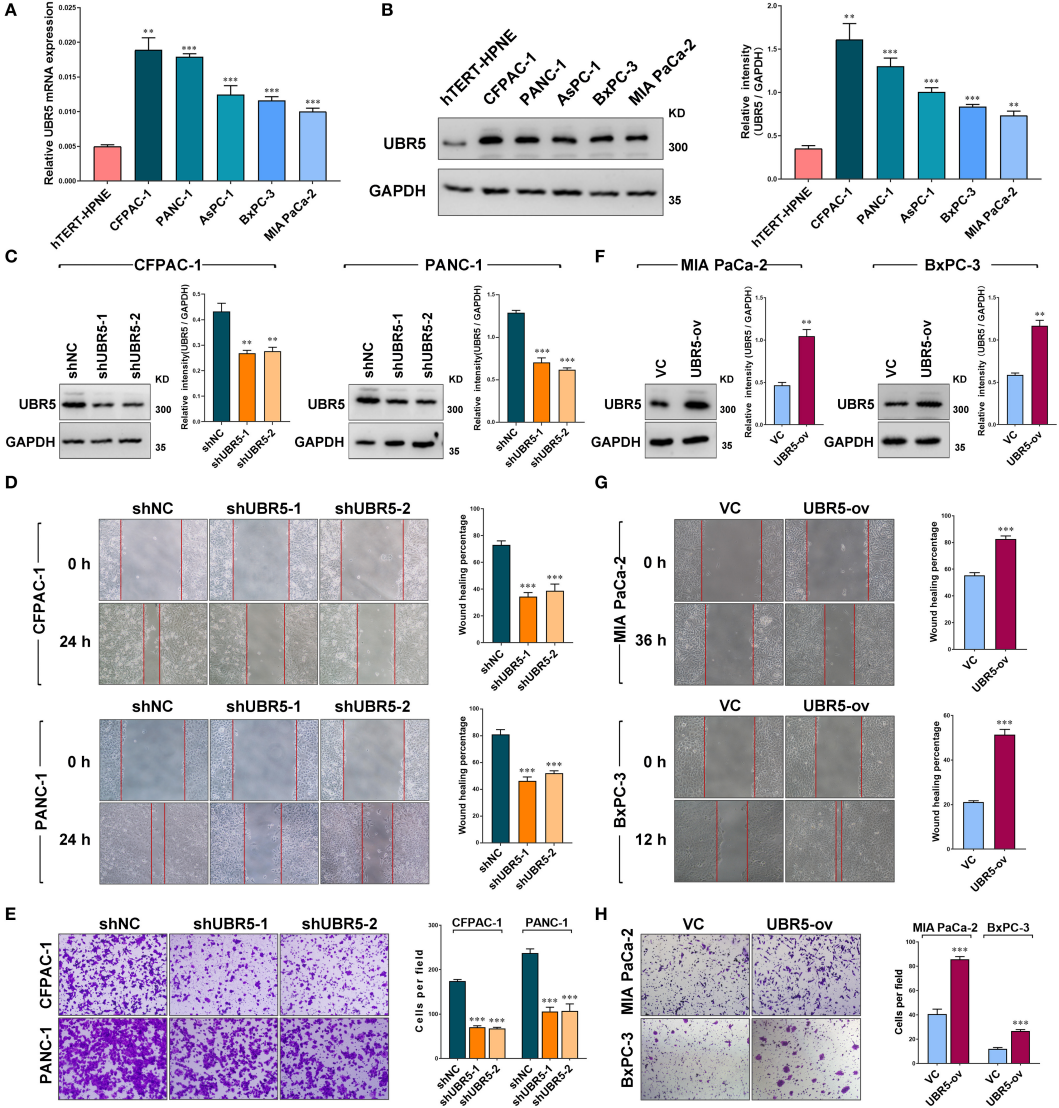
UBR5 promotes pancreatic cancer migration and invasion *in vitro*. **(A)** Relative mRNA expression and **(B)** protein levels of UBR5 in various pancreatic cancer cells and normal pancreatic cells measured by quantitative real-time PCR and western blot separately. The histogram shows the densitometric analysis of the bands. **(C)** The protein levels of UBR5 in CFPAC-1 and PANC-1 cells after infection with shNC or shUBR5. The histogram shows the densitometric analysis of the bands. **(D,E)** Wound healing and invasion assays in CFPAC-1 and PANC-1 cells infected with shNC or shUBR5. **(F)** The UBR5 protein levels in MIA PaCa-2 and BxPC-3 transiently overexpressing UBR5. The histogram shows the densitometric analysis of the bands. **(G,H)** Wound healing and invasion assays in MIA PaCa-2 and BxPC-3 transiently overexpressing UBR5. Data represents mean ± SEM (n = 3 independent biological repeats). **, *P* < 0.01; ***, *P* < 0.001.

Consistent with the observation that UBR5 was upregulated in pancreatic cancer tissues, all five pancreatic cancer cell lines (AsPC-1, BxPC-3, CFPAC-1, MIA PaCa-2, and PANC-1) we tested exhibited significantly higher UBR5 expression at both mRNA and protein levels than the normal human pancreatic duct epithelial hTERT-HPNE cells (Figures 2A,B). In order to investigate its functional role, we knocked down endogenous UBR5 *via* lentivirus-mediated shRNAs in CFPAC-1 and PANC-1 cells (Figure 2C), which presented relative high expression of UBR5 (Figures 2A,B). Interestingly, the wound healing assay and matrigel-based transwell invasion assay showed that UBR5 knockdown resulted in a dramatic decrease in both the migration and invasion of CFPAC-1 and PANC-1 cells *in vitro* (Figures 2D,E). Conversely, overexpression of UBR5 in MIA PaCa-2 and BxPC-3 cells (Figure 2F), which had relatively low intrinsic expression of UBR5 among the five tested pancreatic cancer cell lines (Figures 2A,B), significantly enhanced their migratory and invasive ability *in vitro* (Figures 2G,H), indicating that UBR5 could promote the migration and invasion of pancreatic cancer cells.

### UBR5 Interacts With and Destabilizes CAPZA1

**Figure 3:**
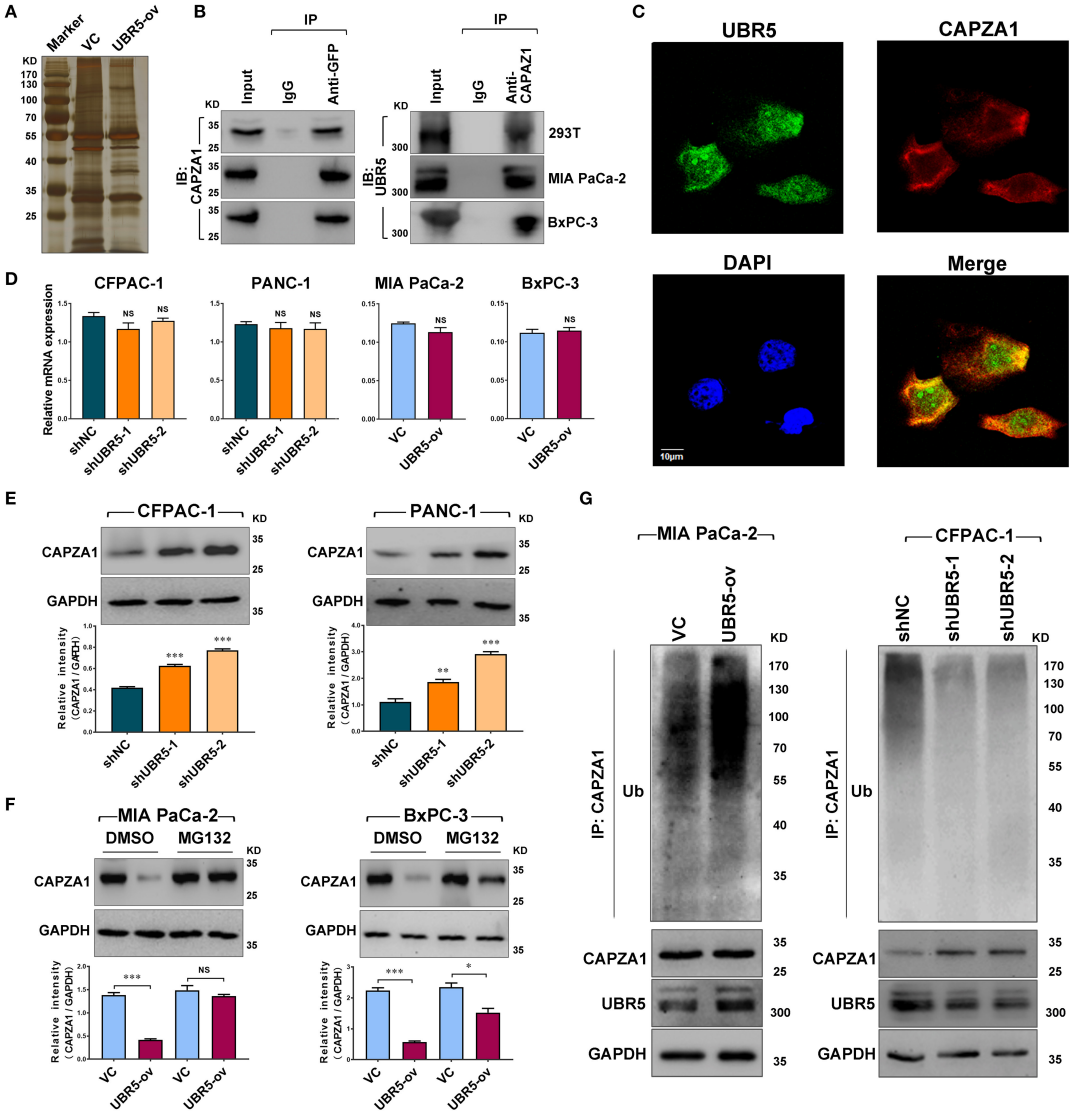
UBR5 interacts with and destabilizes CAPZA1. **(A)** Immunoprecipitation and the silver staining assay in UBR5-binding proteins. The vector control (VC) or GFP-tagged UBR5 (UBR5-ov) plasmid was transfected into HEK-293T cells, and anti-GFP antibody was used to immunoprecipitate UBR5-binding proteins. The immunoprecipitates were separated on SDS-PAGE and stained by silver staining. **(B)** The associations between UBR5 and CAPZA1. HEK-293T, MIA PaCa-2 and BxPC-3 cells were transfected with the plasmid encoding GFP-tagged UBR5 and immunoprecipitation was carried out for the detection of the associations between UBR5 and CAPZA1. **(C)** The cellular co-localization of UBR5 and CAPZA1 in CFPAC-1 cells. Scale bar = 10μm. **(D)** The relative mRNA expression of CAPZA1 in CFPAC-1, PACN-1 cells infected with shUBR5 and in MIA PaCa-2, BxPC-3 transiently overexpressing UBR5. **(E)** The protein level of CAPZA1 assessed by western blot in CFPAC-1 and PANC-1 cells infected with shUBR5 or shNC. **(F)** The relative CAPZA1 protein levels in UBR5-overexpressing MIA PaCa-2 and BxPC-3 treated with DMSO or MG132. **(G)** The ubiquitination level of CAPZA1 in UBR5-overexpressing MIA PaCa-2 cells or UBR5-knockdown CFPAC-1 cells treated with MG132. Data represents mean ± SEM (n = 3 independent biological repeats). NS, not significant; *, *P* < 0.05; **, *P* < 0.01; ***, *P* < 0.001.

To identify the potential UBR5-binding proteins, we performed co-immunoprecipitation assays using the anti-GFP antibody in HEK-293T cells transfected with vector control or GFP-tagged UBR5 plasmid. Total immunoprecipitates (Figure 3A) were then subjected to the mass spectrometry analysis. The potential interacting proteins for UBR5 were selected based on the following 2 criteria: (1) unique peptides of proteins were more than 2; and (2) immunoprecipitates only existed in the GFP-tagged UBR5 group in comparison with those obtained from vector control-transfected cells. As a result, 30 potential UBR5-interacting proteins were identified (Supplementary Table 4). Interestingly, more than one fourth (8 from 30) of them were related to actin cytoskeleton organization. Among them, F-actin-capping protein subunit alpha-1 (CAPZA1) has recently been related to the migratory and invasive abilities of cancer cells. We therefore focused on CAPZA1 to explore the mechanism of UBR5-induced enhancement of pancreatic cancer cells migration and invasion. Using reciprocal Co-IP assays, the interaction of UBR5 with CAPZA1 was confirmed not only in HEK-293T cells but also in pancreatic cancer MIA PaCa-2 and BxPC-3 cells transfected with GFP-tagged UBR5 (Figure 3B). However, in the control cells, which do not express GFP-UBR5 fusion proteins, CAPZA1 protein could not be detected in the immunoprecipitates pulled down by anti-GFP antibody (Supplementary Figure 2). Moreover, the confocal microscopy showed the co-localization of UBR5 and CAPZA1 in pancreatic cancer cells (Figure 3C), further supporting the interaction between these two proteins.

We next investigated the influence of UBR5 on CAPZA1 expression in pancreatic cancer cells. Although knockdown or overexpression of UBR5 had no impact on the mRNA levels of CAPZA1 (Figure 3D), its protein levels were negatively regulated by UBR5 (Figures 3E,F). Inhibition of the proteasome by the specific inhibitor MG132 largely reversed the downregulation of CAPZA1 induced by UBR5 overexpression (Figure 3G). We also overexpressed UBR5 in the normal human pancreatic ductal epithelial hTERT-HPNE cells. Although UBR5 overexpression significantly downregulated the expression of CAPZA1 in hTERT-HPNE cells, it failed to promote their *in vitro* migratory and invasive ability (Supplementary Figure 3). Meanwhile, the accumulation of ubiquitinated CAPZA1 were observed in UBR5-overexpressing MIA PaCa-2 cells in the presence of MG132 (Figure 3H). In contrast, the ubiquitinated CAPZA1 were reduced in UBR5-knockdown CFPAC-1 cells (Figure 3H). These results indicate that UBR5 destabilizes CAPZA1 proteins via the ubiquitin-proteasome pathway in pancreatic cancer cells.

### UBR5 Promotes Pancreatic Cancer Migration and Invasion *via* CAPZA1-Mediated F-Actin Remodeling

**Figure 4:**
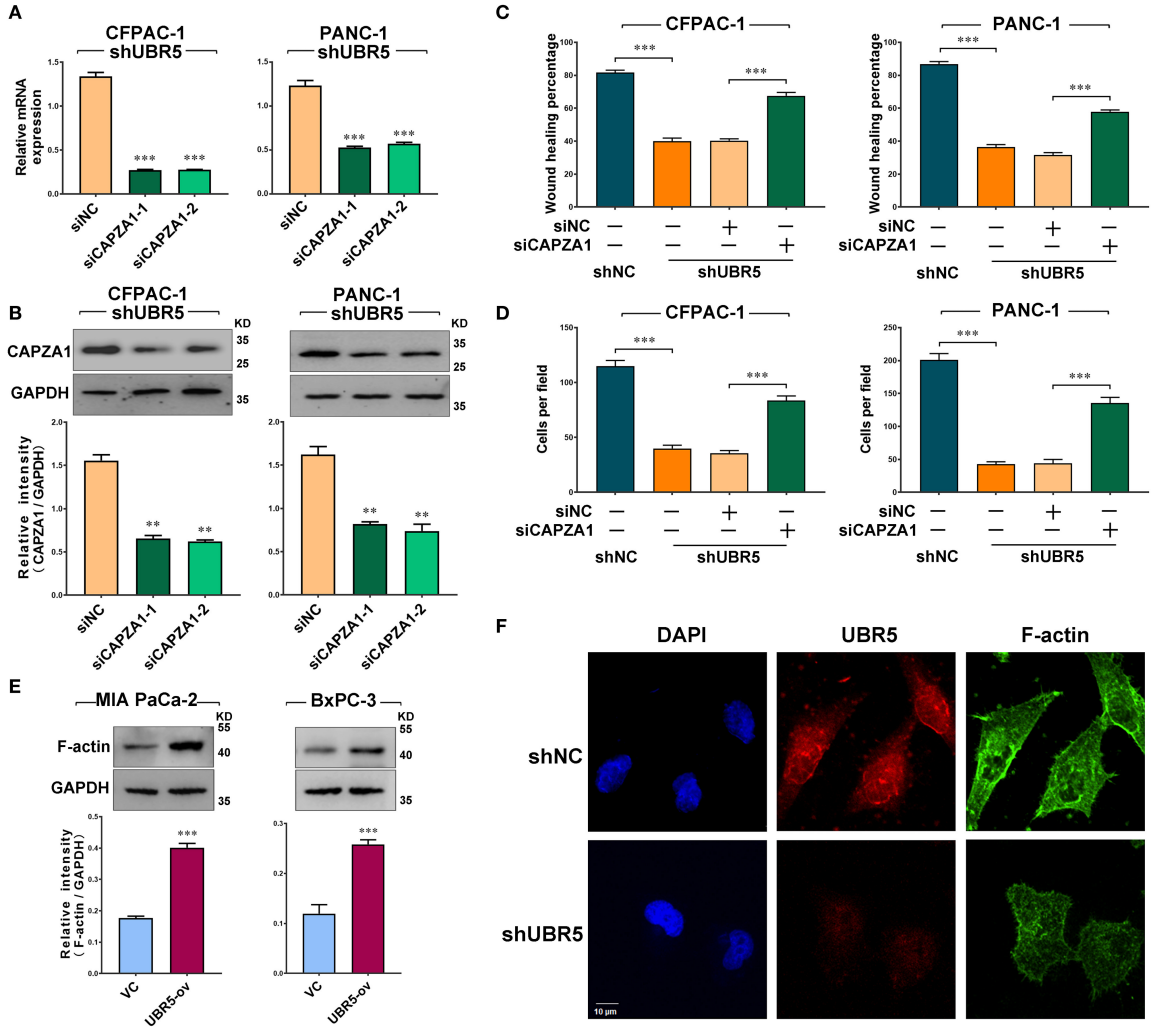
UBR5 promotes pancreatic cancer migration and invasion via CAPZA1-mediated F-actin remodeling. **(A)** CAPZA1 mRNA expression in CFPAC-1 and PANC-1cells infected with shUBR5. **(B)** Western blot analysis of CAPZA1 protein levels in CFPAC-1 and PANC-1 cells infected with shUBR5. **(C)** Wound healing assays in CFPAC-1 and PANC-1 cells transfected with shNC, shUBR5, shUBR5 and siNC, shUBR5 and siCAPZA1. **(D)** Invasion assays in CFPAC-1 and PANC-1 cells transfected with shNC, shUBR5, shUBR5 and siNC, shUBR5 and siCAPZA1 respectively. **(E)** F-actin protein levels in MIA PaCa-2 and BxPC-3 cells overexpressing UBR5. **(F)** Immunofluorescence staining of F-actin and UBR5 in CFPAC-1 cells transfected with shNC or shUBR5. Scale bar = 10μm. Data represents mean ± SEM (n = 3 independent biological repeats unless otherwise indicated). **P < 0.01, ***P < 0.001.

Based on the above findings, we next investigated the role of CAPZA1 in pancreatic cancer cells. Given that CAPZA1 was markedly upregulated in UBR5-knockdown cells (Figure 3E), we downregulated CAPZA1 by siRNAs in these cells (Figures 4A,B). As shown in Figures 4C,D and Supplementary Figure 4, the UBR5 knockdown-induced inhibition of cell migration and invasion could be largely reversed by siCAPZA1 in CFPAC-1 and PANC-1 cells. The decreased level of CAPZA1 could induce the accumulation of actin filament (F-actin) and promote its dynamic remodeling, which offered the impetus for cell invasion and migration. As expected, the increase of F-actin intensity was observed in pancreatic cancer cells transfected with UBR5 plasmid (Figure 4E). Additionally, we performed immunofluorescence staining of F-actin in UBR5-knockdown CFPAC-1 cells. Compared with the control cells, the levels of F-actin were significantly decreased in UBR5-knockdown cells. It was also showed that F-actin formation was suppressed by UBR5 deficiency (Figure 4F), suggesting that UBR5 enhanced pancreatic cancer migration and invasion via promoting CAPZA1 degradation-induced F-actin remodeling in pancreatic cancer cells.

### UBR5 Deficiency Reduces Pancreatic Cancer Metastasis *in vivo*

**Figure 5:**
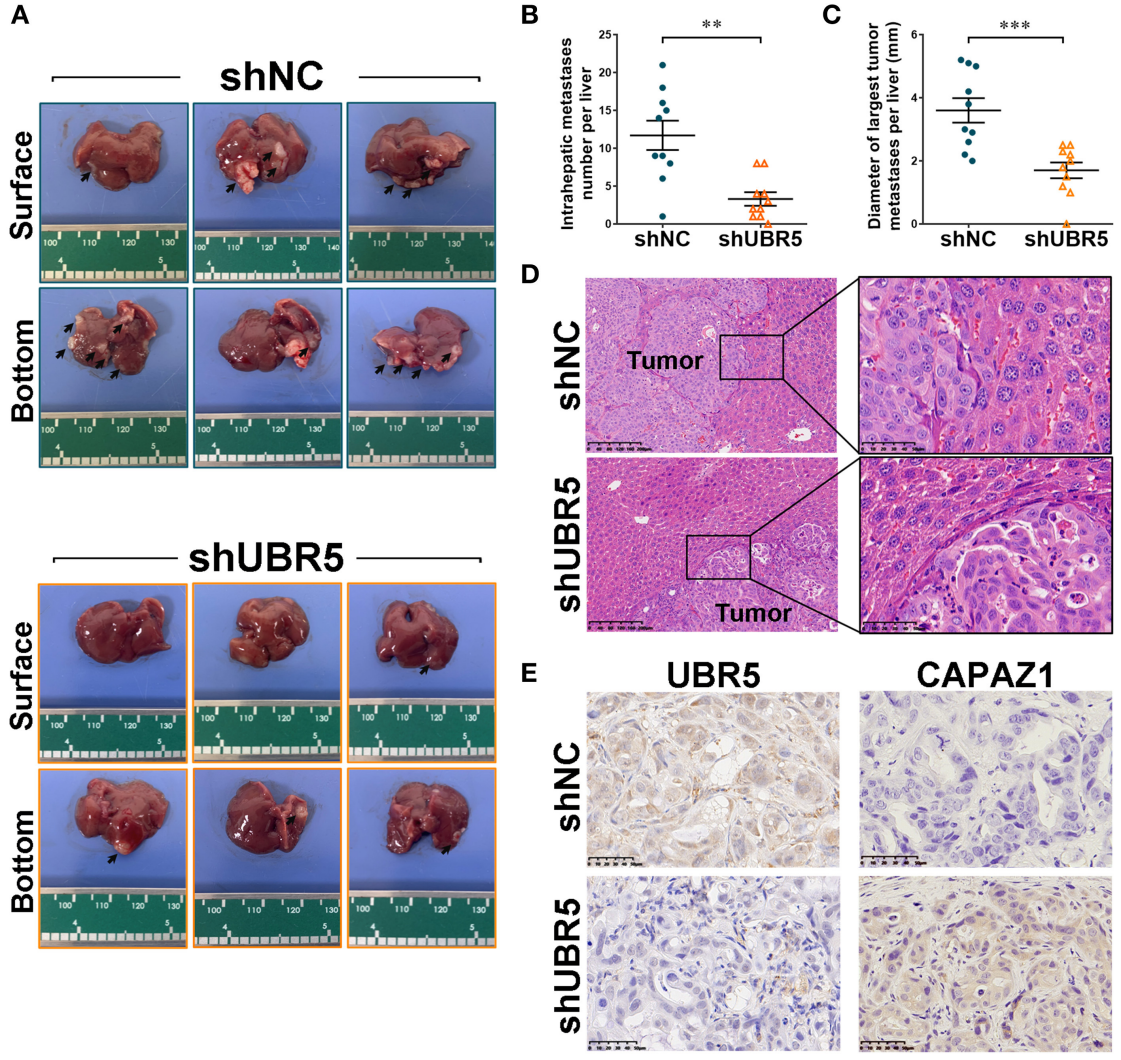
UBR5 deficiency reduces pancreatic cancer metastasis *in vivo*. **(A)** Representative images of liver-metastatic lesions from nude mice after injection of CFPAC-1 cells infected with shNC or shUBR5 (n=10 for each group). **(B)** The number of lesions in shNC and shUBR5 groups. **(C)** The diameter of the largest metastatic tumor per liver was measured. **(D)** H&E staining of livers with the metastatic lesions. Scale bar of left panels: 200μm; Scale bar of right panels: 50μm. **(E)** IHC staining of UBR5 and CAPZA1 protein expression in the metastatic tumors. Scale bar: 50μm. Data represents mean ± SEM (n = 3 independent biological repeats unless otherwise indicated). **P < 0.01, ***P < 0.001.

To further assess the role of UBR5 *in vivo*, the CFPAC-1 cells infected with shUBR5 were injected into the spleen of nude mice to establish the liver-metastasis models. Six weeks later, these nude mice were sacrificed and the micro-hepatoma lesions in livers were measured. It was found that livers from the mice injected with CFPAC-1 cells infected with shUBR5 were covered with less micro-hepatoma lesions, compared with mice processed with control cells (Figure 5A). We calculated the number of intrahepatic metastatic tumors and the diameter of largest intrahepatic metastatic tumors, and it was observed that UBR5 knockdown led to significantly decreased number and size of liver metastatic nodules in mice (Figures 5B,C). The metastatic nodules in livers were confirmed by histopathological examination (Figure 5D). In addition, we performed the immunohistochemistry analyses of UBR5 and CAPZA1 in metastatic tumors. As shown in Figure 5E, the low level of UBR5 was associated with the increased level of CAPZA1 in UBR5-knockdown tumors. These *in vivo* data were consistent with our *in vitro* findings, supporting a metastasis-promoting effect of UBR5 *via* destabilizing CAPZA1 in pancreatic cancer.

## Discussion

**Figure 6:**
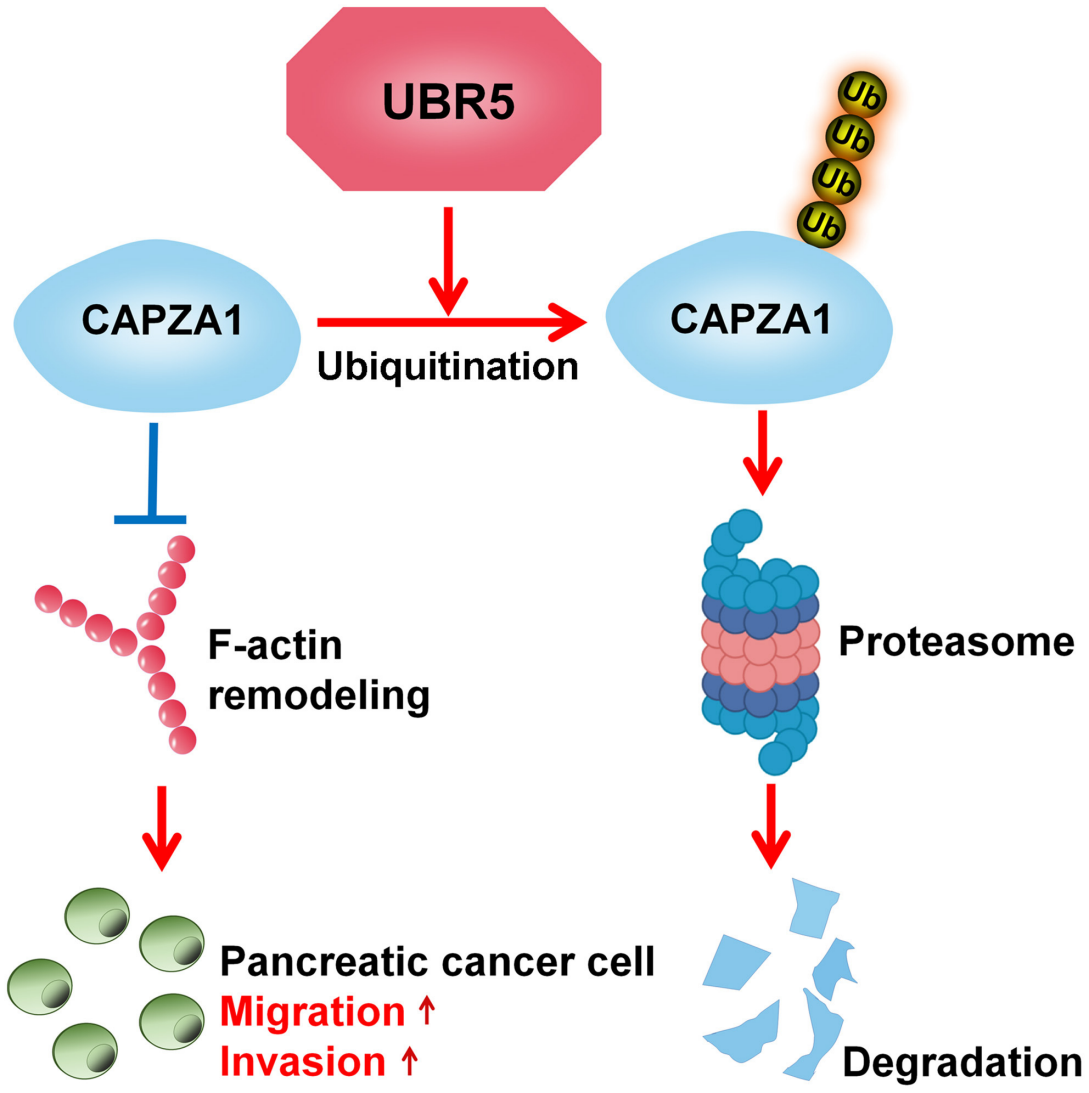
Schematic diagram of UBR5 promoting pancreatic cancer metastasis. A model depicting the role of UBR5 in pancreatic cancer. E3 ubiquitin ligase UBR5 mediates the ubiquitin-proteasome-dependent degradation of CAPZA1, which in turn enhances pancreatic cancer metastasis via promoting F-actin remodeling.

In recent decades, increasing attention has been paid to the roles of UPS in the processes of oncogenesis and cancer development (28). Understanding the basis of pancreatic cancer from the UPS perspective will provide novel insights into this disease. Here, we advance the functional role of the E3 ubiquitin ligase UBR5, a vital regulator of the UPS, in pancreatic cancer and provide solid evidence demonstrating that UBR5 could remarkably promote pancreatic cancer metastasis. With respect to the mechanisms, our study identified CAPZA1 as a novel substrate of UBR5 and suggested that UBR5 mediated the ubiquitin-proteasome-dependent degradation of CAPZA1, which in turn facilitated pancreatic cancer metastasis via promoting F-actin remodeling (Figure 6).

UBR5 is emerging as a key role in cancer development and progression. Nevertheless, its role in pancreatic cancer was rarely studied. A recent study indicated the growth-promoting effect of UBR5 on pancreatic cancer cells (29). Here, we extended the understanding of its function to the regulation of pancreatic cancer metastasis. Based on the loss-of-function and gain-of-function studies, UBR5 was shown to contribute to the metastasis of pancreatic cancer. Our functional findings were supported by the clinical observation that pancreatic cancer patients with high tumoral expression of UBR5 exhibited more metastasized lymph nodes. High propensity for metastasis is the leading cause of adverse prognosis and extremely high mortality in pancreatic cancer patients (4). Unexpectedly, high UBR5 level was also significantly associated with worse prognosis of pancreatic cancer patients. It is worthy to note that the metastasis-promoting role of UBR5 is not restricted to pancreatic cancer. Data from Oncomine and CPTAC databases unveiled that UBR5 overexpression was common in various cancer types such as gastric and breast cancers. The high expression of UBR5 has been associated with increased invasive behavior of both gastric cancer and breast cancer (17, 30), suggesting that the metastasis-promoting property of UBR5 may be widely present in cancers.

UBR5 is indicated to mainly exist in the nucleus, and the previous studies on UBR5 have largely focused on its regulation of nuclear proteins (31). UBR5 was found to localize to damaged DNA double strand breaks and interacted with OTUD5 deubiquitinase to regulate transcription at damaged chromatin (32). In addition, UBR5 could accelerate the ubiquitination of nuclear myosin 1c to regulate transcription in early G1 cells (14). However, recent reports revealed that UBR5 was present not only in the nucleus but also in the cytoplasm (33). UBR5 could localized to the centrosomal periphery and its interaction with centrosomal component was required for cytoplasmic organization of centriolar satellites (34). In our study, the cytoplasmic location of UBR5 was also observed in pancreatic cancer cells and multiple cytoplasmic proteins were identified as potential UBR5-binding proteins by mass spectrometry. Our results, together with the previous findings, indicate that the interaction and regulation of cytoplasmic proteins is also important for UBR5 to exert its biological function.

This study, for the first time, described the cytoplasmic protein CAPZA1, an actin capping protein, as an important substrate of UBR5 and an important mediator of UBR5-induced cell migration and invasion in pancreatic cancer. CAPZA1 has been involved in cancer metastasis previously. It has been reported that, in gastric and liver cancer, the expression of CAPZA1 was significantly decreased, which contributed to an increased metastatic ability of cancer cells (35–37). Our findings suggested that CAPZA1 might function as a metastasis suppressor downstream of UBR5. The reduced migration and invasion ability induced by knockdown of UBR5 in pancreatic cancer cells was largely abolished by the introduction of siCAPZA1, indicating the therapeutic potential of strategies to disrupt the interaction between UBR5 and CAPZA1 for preventing cancer metastasis especially in patients with high UBR5 expression. Intriguingly, more than one fourth of potential interacting proteins identified using IP-MS analyses for UBR5 were related to actin cytoskeleton organization. It would be interesting to investigate the regulatory effect of UBR5 on other cytoskeleton organization-related proteins in the future.

The regulation of F-actin remodeling has been described as an important mechanism by which CAPZA1 influenced cancer metastasis (37). F-actin, formed by the polymerization of actin monomers (globular or G-actin), is considered to be a critical player in cell morphogenesis and cell motility (38). Actin-binding proteins like CAPZA1 disassemble from the end of F-actin, leading to the decrease in G-actin and the accumulation of F-actin. The actin-binding protein deficiency-induced actin cytoskeleton remodeling, reflected by the increase intracellular level of F-actin, has been shown to facilitate the invasion as well as metastasis of liver cancer cells (39). In our study, it was observed that the decrease in CAPZA1 was associated with marked increase in F-actin intensity in UBR5-overexpression pancreatic cancer cells that exhibited enhanced migratory and invasion ability. Given that CAPZA1 functions as the substrate of UBR5, we can reasonably speculate that CAPZA1 degradation-induced actin reorganization might contribute to UBR5-induced pancreatic cancer cells metastasis. It has been regarded that the reorganization of actin cytoskeleton drove epithelial-mesenchymal transition (EMT) of cancer cells (38, 40). Further studies are warranted to investigate whether the elevated UBR5 expression can induce EMT of pancreatic cancer cells.

In summary, the current study provides evidence demonstrating UBR5 as a potent metastasis promoter for pancreatic cancer and suggests that UBR5 exerts its metastasis-promoting effect via promoting the ubiquitination and degradation of CAPZA1. Therefore, targeting UBR5 or UBR5-mediated CAPZA1 ubiquitination will provide promising strategies for preventing cancer metastasis in pancreatic cancer patients, particularly in those with high UBR5 expression.

## Data Availability Statement

The original contributions presented in the study are included in the article/Supplementary Material, further inquiries can be directed to the corresponding author/s.

## Ethics Statement

The studies involving human participants were reviewed and approved by the Research Ethics Committee of Taizhou Hospital. The patients/participants provided their written informed consent to participate in this study. The animal study was reviewed and approved by the Medical Experimental Animal Care Commission at the Shanghai Cancer Institute.

## Author Contributions

YG and HT conceived the study and designed the experiments. JL and WZ performed experiments, analyzed data, and wrote the manuscript. JG, MD, HL, ML, and YF performed some experiments. YL, DZ, GX, and HC assisted the writing and analyzed data. MY and XM contributed to the revision of manuscript. All authors reviewed the results and approved the final version of the manuscript.

## Conflict of Interest

The authors declare that the research was conducted in the absence of any commercial or financial relationships that could be construed as a potential conflict of interest. The handling editor declared a shared affiliation, though no other collaboration, with several of the authors JL, WZ, JG, MD, HL, ML, HC, YL, DZ, GX, XM, MY, HT, and YG.
