# Corrigendum: E3 Ubiquitin Ligase UBR5 Promotes the Metastasis of Pancreatic Cancer *via* Destabilizing F-Actin Capping Protein CAPZA1

**DOI:** 10.3389/fonc.2021.693855

**Published:** 2021-05-11

**Authors:** Jin Li, Wei Zhang, Jian Gao, Min Du, Huimin Li, Mengge Li, Hui Cong, Yuan Fang, Yiyi Liang, Dan Zhao, Gang Xiang, Xiaojing Ma, Ming Yao, Hong Tu, Yu Gan

**Affiliations:** ^1^ State Key Laboratory of Oncogenes and Related Genes, Shanghai Cancer Institute, Renji Hospital, Shanghai Jiao Tong University School of Medicine, Shanghai, China; ^2^ Department of Medical Oncology, The First Affiliated Hospital of USTC, Division of Life Sciences and Medicine, University of Science and Technology of China, Hefei, China; ^3^ Organ Transplantation Center, The First Affiliated Hospital of Kunming Medical University, Kunming Medical University, Kunming, China; ^4^ State Key Laboratory of Microbial Metabolism, Sheng Yushou Center of Cell Biology and Immunology, School of Life Science and Biotechnology, Shanghai Jiao Tong University, Shanghai, China

**Keywords:** UBR5, metastasis, CAPZA1, ubiquitination, pancreatic cancer

In the original article, there were two mistakes in **Figure 2** as published. In **Figure 2D**, the image for 0h shNC group was same as the image for 0h shUBR5-1 group in the wound healing experiment of PANC-1 cells. In **Figure 2E**, the cell line names above the bar graph (right panel) were incorrectly given as “CFPAC-1” and “BxPC-3”. They should be “CFPAC-1” and “PANC-1”, consistent with the cell line names given in the left panel. The corrected **Figure 2** appears below.

**Figure 2:**
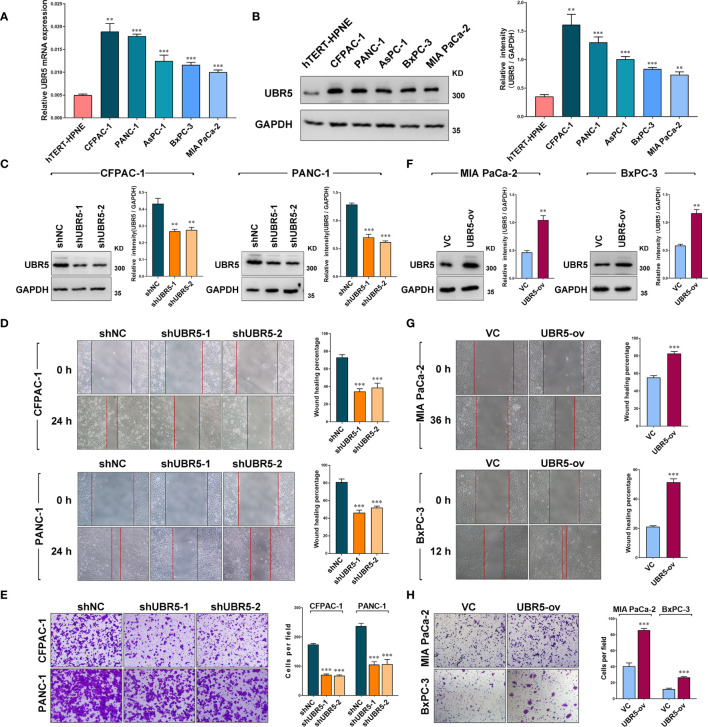
UBR5 promotes pancreatic cancer migration and invasion in vitro. **(A)** Relative mRNA expression and **(B)** protein levels of UBR5 in various pancreatic cancer cells and normal pancreatic cells measured by quantitative real-time PCR and western blot separately. The histogram shows the densitometric analysis of the bands. **(C)** The protein levels of UBR5 in CFPAC-1 and PANC-1 cells after infection with shNC or shUBR5. The histogram shows the densitometric analysis of the bands.** (D, E)** Wound healing and invasion assays in CFPAC-1 and PANC-1 cells infected with shNC or shUBR5. **(F)** The UBR5 protein levels in MIA PaCa-2 and BxPC-3 transiently overexpressing UBR5. The histogram shows the densitometric analysis of the bands. **(G, H)** Wound healing and invasion assays in MIA PaCa-2 and BxPC-3 transiently overexpressing UBR5. Data represents mean ± SEM (n = 3 independent biological repeats). **P<0.01; ***P<0.001.

The authors apologize for these errors and state that this does not change the scientific conclusions of the article in any way. The original article has been updated.

