# Supplementary material for: E3 Ubiquitin Ligase UBR5 Promotes the Metastasis of Pancreatic Cancer via Destabilizing F-Actin Capping Protein CAPZA1
Source: Front Oncol. 2021 Mar 12;11:634167. doi: 10.3389/fonc.2021.634167 (PMC7994773; doi:10.3389/fonc.2021.634167)
Supplement: Supplementary file 1 [file Data_Sheet_1.ZIP › supplementary figures +response/Supplementary Figure legend.docx]

**Supplementary Figure Legends**

**Figure S1. UBR5 mRNA expression in multiple cancers. (A)** The mRNA expression of UBR5 in cancers and normal tissues (from the Oncomine database). Red color indicated over-expression while blue indicated under-expression (fold change ≥ 1.5). The number of each cell represented the amount of significant unique analyses (*P* < 0.05). **(B)** The protein levels of UBR5 in cancer and normal tissues (from CTPAC). Red color indicates the tumor samples while blue indicates the normal tissues.

**Figure S2.** **The Co-IP assays in pancreatic cancer cells transfected with vector control or GFP-tagged UBR5 plasmid.** The vector control (VC) or GFP-tagged UBR5 (UBR5-ov) plasmid was transfected into MIA PaCa-2 cells and BxPC-3 cells and the anti-GFP immunoprecipitates were subjected to western blotting analysis using anti-CAPZA1 primary antibody.

**Figure S3.** **The role of UBR5 in hTERT-HPNE cells.** **(A)** The protein levels of UBR5 and CAPZA1 were assessed by western blot in UBR5-overexpressing hTERT-HPNE cells. The wound healing **(B)** and invasion **(C)** assays were carried out to analyze the *in vitro* migratory and invasive ability of UBR5-overexpressing hTERT-HPNE cells.

**Figure S4.** ***In vitro* migratory and invasive assays in CFPAC-1 and PANC-1 cells.** CFPAC-1 and PANC-1 cells were transfected with shNC, shUBR5, shUBR5 and siNC, shUBR5 and siCAPZA1, respectively. Representative images of wound healing **(A)** and invasion **(B)** assays were shown here.
