# Supplementary figures and images for: E3 Ubiquitin Ligase UBR5 Promotes the Metastasis of Pancreatic Cancer via Destabilizing F-Actin Capping Protein CAPZA1
Source: Front Oncol. 2021 Mar 12;11:634167. doi: 10.3389/fonc.2021.634167 (PMC7994773; doi:10.3389/fonc.2021.634167)

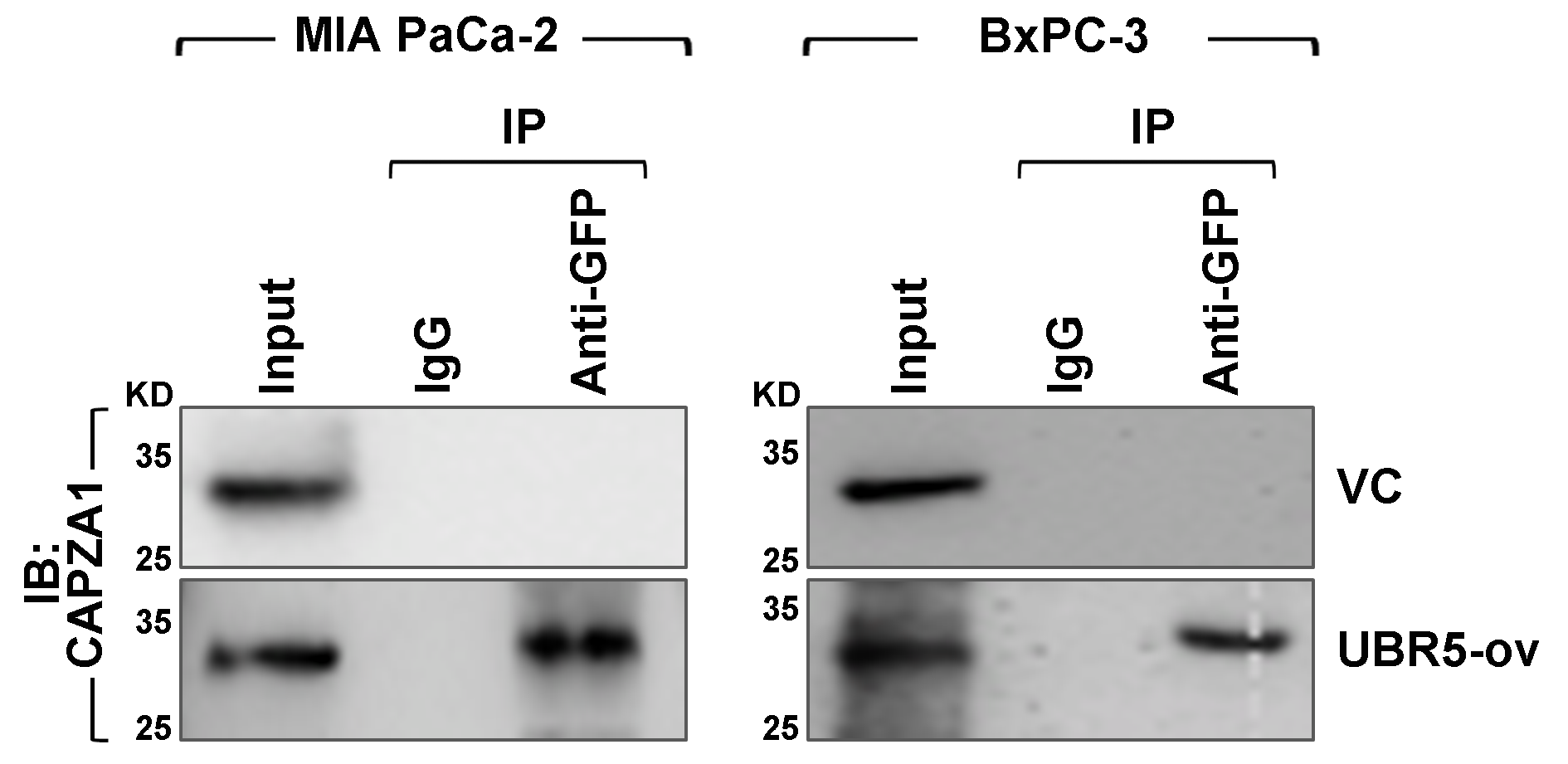

Supplement: Supplementary file 1 [file Data_Sheet_1.ZIP › supplementary figures +response/Sup Fig S2.tif]

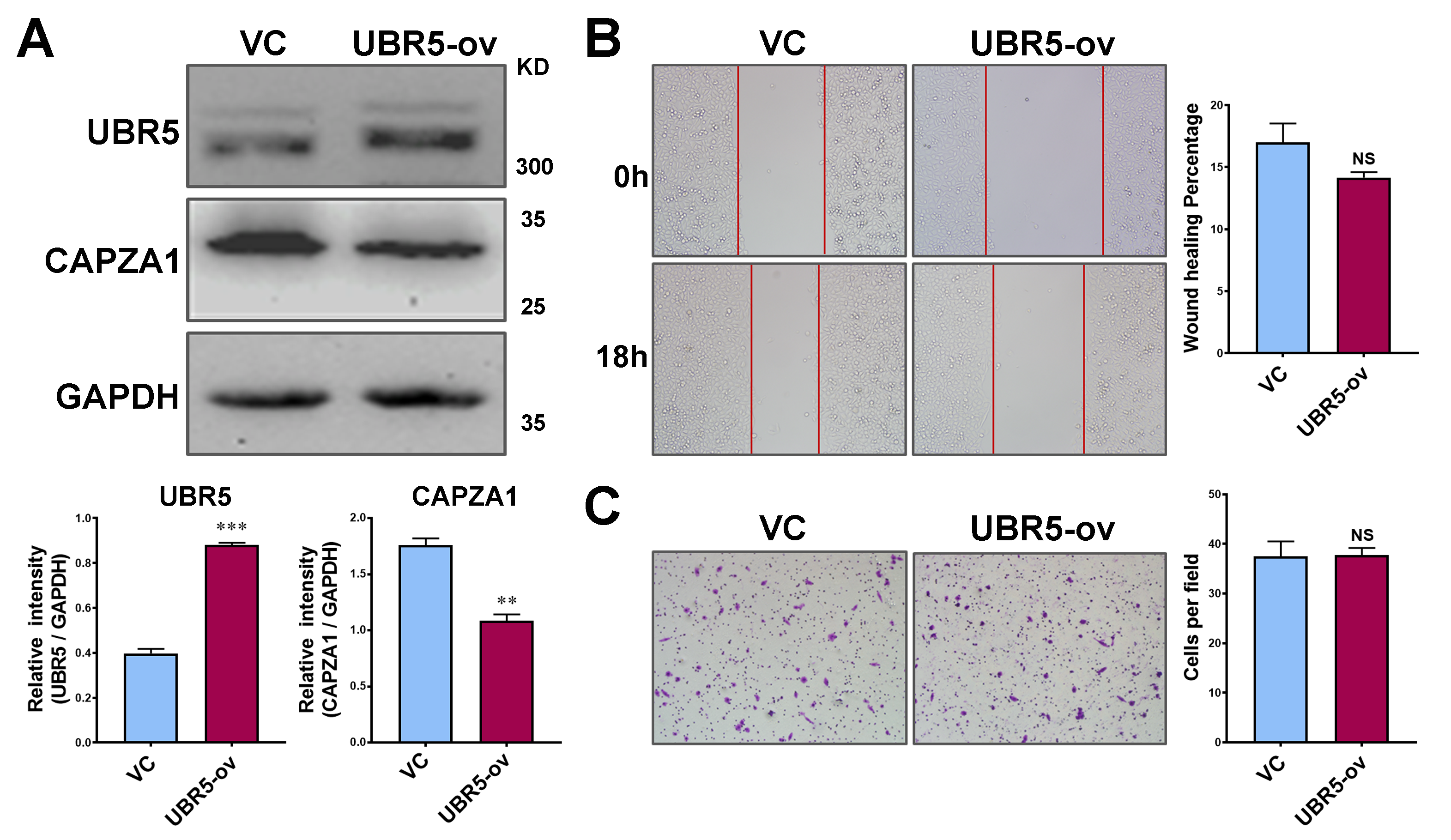

Supplement: Supplementary file 1 [file Data_Sheet_1.ZIP › supplementary figures +response/Sup Fig S3.tif]

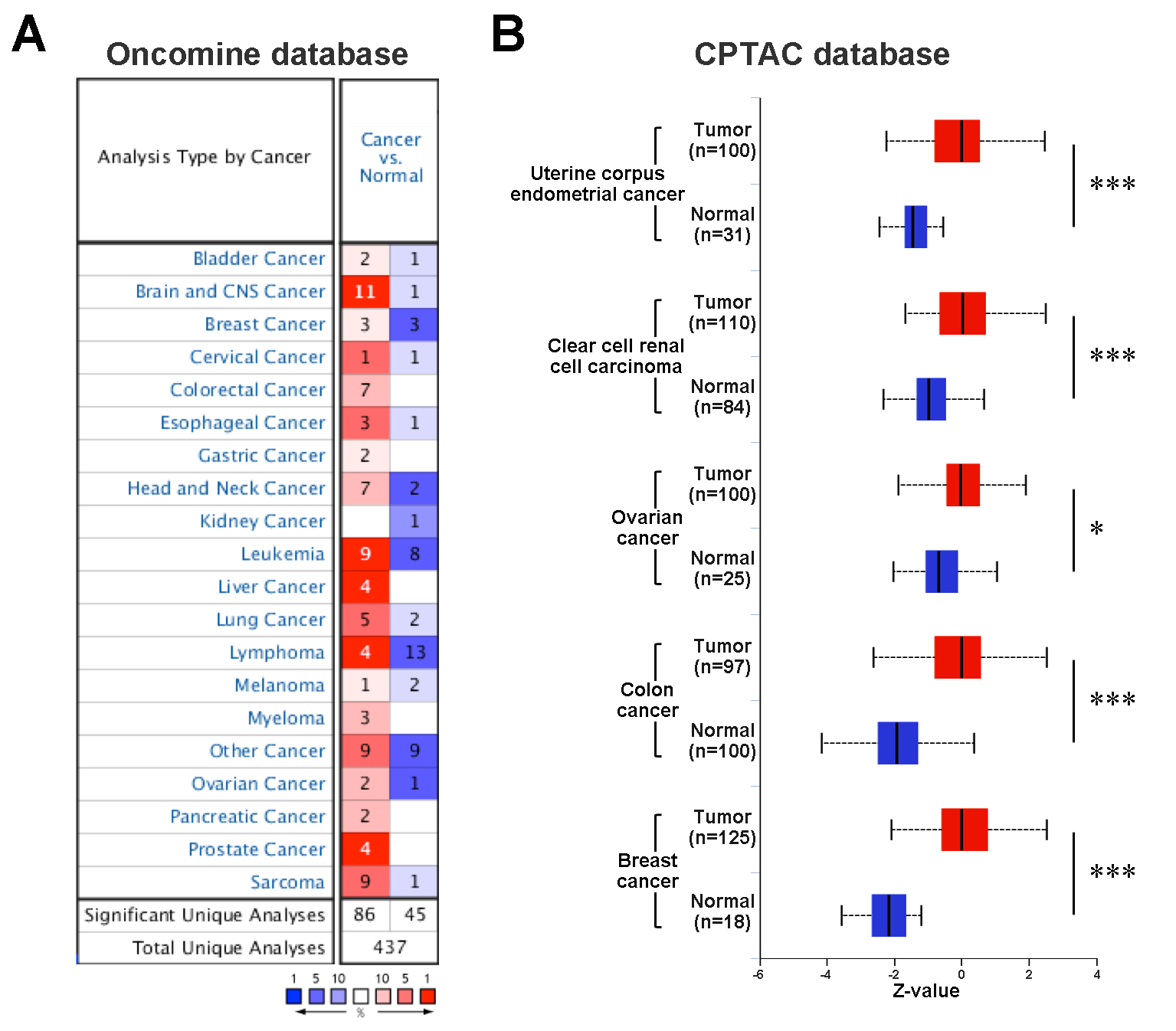

Supplement: Supplementary file 1 [file Data_Sheet_1.ZIP › supplementary figures +response/Sup Fig S1.tif]

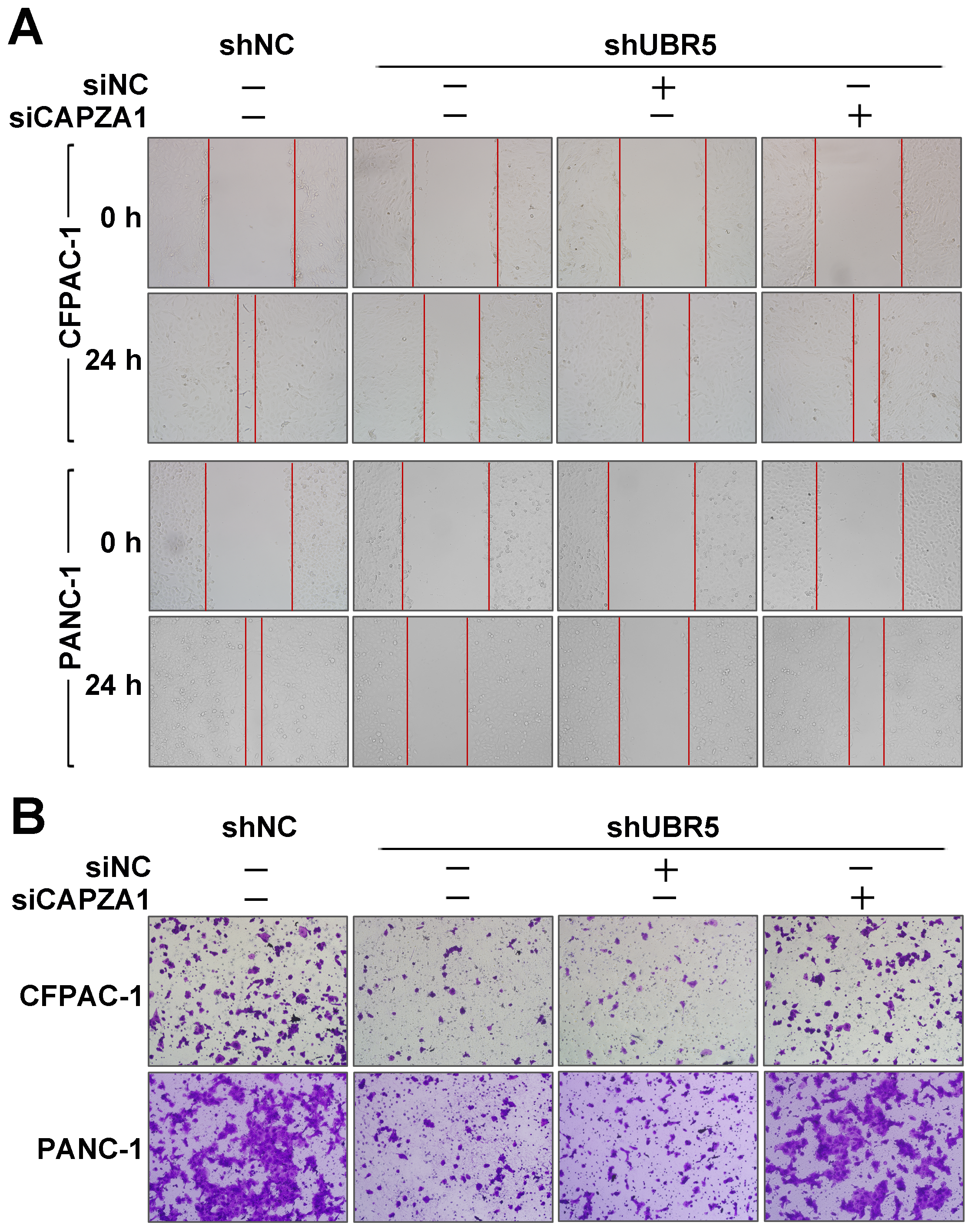

Supplement: Supplementary file 1 [file Data_Sheet_1.ZIP › supplementary figures +response/Sup Fig S4.tif]
