# Supplementary material for: E3 Ubiquitin Ligase UBR5 Promotes the Metastasis of Pancreatic Cancer via Destabilizing F-Actin Capping Protein CAPZA1
Source: Front Oncol. 2021 Mar 12;11:634167. doi: 10.3389/fonc.2021.634167 (PMC7994773; doi:10.3389/fonc.2021.634167)
Supplement: Supplementary file 2 [file Data_Sheet_2.ZIP › Supplemetary tables + Response/Supplementary Table2 Primers used in this study..docx]

**Table S2. Primers for the quantitative real-time PCR**

| Name | Primer Sequence (5’-3’) |
| --- | --- |
| H-UBR5-F | GTCCATCCATTTCGTGGTTCA |
| H-UBR5-R | CCAATTCCAATCTGTCTGGCTG |
| H-CAPZA1-F  H-CAPZA1-R | GCCGACTTCGATGATCGTGT  AGTAGCCGAACGTCATGAATAC |
| H-GAPDH-F | AGAAGGCTGGGGCTCATTTG |
| H-GAPDH-R | AGGGGCCATCCACAGTCTTC |
