# Supplementary material for: E3 Ubiquitin Ligase UBR5 Promotes the Metastasis of Pancreatic Cancer via Destabilizing F-Actin Capping Protein CAPZA1
Source: Front Oncol. 2021 Mar 12;11:634167. doi: 10.3389/fonc.2021.634167 (PMC7994773; doi:10.3389/fonc.2021.634167)
Supplement: Supplementary file 2 [file Data_Sheet_2.ZIP › Supplemetary tables + Response/Supplementary Table3 The antibodies used in this study.docx]

**Table S3. The antibodies for IHC, Co-IP, ICC, WB**

| Antibody | Source | Catalogue number | Dilution | Application | Company |
| --- | --- | --- | --- | --- | --- |
| **Primary antibodies for IHC, Co-IP, ICC, WB** | | | | | |
| UBR5 | Rabbit IgG | Ab175810 | 1:500 | IHC | Abcam |
| CAPZA1  UBR5  CAPZA1  UBR5  CAPZA1  UBR5  CAPZA1  Ubiquitin  F-actin  GAPDH | Mouse IgG  Rabbit IgG  Rabbit IgG  Rabbit IgG  Mouse IgG  Rabbit IgG  Rabbit IgG  Mouse IgG  Mouse IgG  Mouse IgG | 66066-Ig  Ab175810  11806-1-AP  Ab175810  66066-Ig  Ab175810  11806-1-AP  P4D/3936  Ab130935  97166 | 1:100  4 ug/IP  3.5ug/IP  1:200  1:100  1:1500  1:500  1:1000  1:500  1:4000 | IHC  Co-IP  Co-IP  ICC  ICC  WB  WB  WB  WB  WB | Proteintech  Abcam  Proteintech  Abcam  Proteintech  Abcam  Proteintech  CST  Abcam  CST |
| **Secondary antibodies for IHC, ICC, WB** | | | | | |
| (HRP)-conjugated  Alexa Fluor® 488  Alexa Fluor®  647  Anti-Mouse IgG HRP  Anti-Rabbit IgG HRP | Rabbit IgG  Rabbit IgG  Mouse IgG  Goat  Goat | MP-7401  Ab150077  Ab150079  SA00001-2  SA00001-1 | 1:1  1:500  1:500  1:4000  1:3000 | IHC  ICC  ICC  WB  WB | Vector Labaratories  Abcam  Abcam  Proteintech  Proteintech |
