# Supplementary material for: E3 Ubiquitin Ligase UBR5 Promotes the Metastasis of Pancreatic Cancer via Destabilizing F-Actin Capping Protein CAPZA1
Source: Front Oncol. 2021 Mar 12;11:634167. doi: 10.3389/fonc.2021.634167 (PMC7994773; doi:10.3389/fonc.2021.634167)
Supplement: Supplementary file 2 [file Data_Sheet_2.ZIP › Supplemetary tables + Response/Supplementary Table1 Sequences for shUBR5 and siCAPZA1.docx]

**Table S1. Target sequences corresponding to shUBR5 and siCAPZA1**

| Name | Primer Sequence (5’-3’) |
| --- | --- |
| shNC  shUBR5-1  shUBR5-2 | CCGGGCTTCTCCGAACGTGTCACGTCTCGAGACGTGACACGTTCGGAGAAGCTTTTTG  CCGGGCCATTAGAAAGAACCACAAACTCGAGTTTGTGGTTCTTTCTAATGGCTTTTTG  CCGGGCTGTAGATTTCAACTTAGATCTCGAGATCTAAGTTGAAATCTACAGCTTTTTG |
| siNC | TTCTCCGAACGTGTCACGT |
| siCAPZA1-1 | CCACCAGTTTCAGCCTAAA |
| siCAPZA1-2 | CTGCTAAATTCATCACTCA |
